# Using social media advertisement data to monitor the gender gap in STEM: opportunities and challenges

**DOI:** 10.7717/peerj-cs.994

**Published:** 2022-06-21

**Authors:** Reham Al Tamime, Ingmar Weber

**Affiliations:** Hamad Bin Khalifa University (HBKU), Qatar Computing Research Institute, Doha, Qatar

**Keywords:** Advertisement data, Social media, Gender gap in STEM, Interest in STEM, Instagram Ads data, Facebook Ads data, USA, Activity level, Leaky pipeline

## Abstract

Boosting the number of women and girls entering careers involving STEM (Science, Technology, Engineering and Maths) is crucial to achieving gender equality, one of the UN Sustainable Development Goals. Girls and women tend to gravitate away from STEM fields at multiple stages from childhood through mid-career. The leaky pipeline is a metaphor often used to describe the loss of women in STEM and arguably other fields before reaching senior roles. Do interests expressed on social media mirror the leaky pipeline phenomenon? In this article, we collected advertisement data (reach estimates) from Facebook and Instagram disaggregated by US metros, age, gender, and interests related to STEM. We computed the Gender Gap Index (GGI) for each US metro and age group. We found that on Instagram, the GGIs for interest in *Science* decrease as users’ age increases, suggesting that relatively there is evidence that that women, compared to men, are losing interest in STEM at older ages. In particular, we find that on Instagram, there are plausible relative trends but implausible absolute levels. Nevertheless, is this enough to conclude that online data available from Instagram mirror the leaky pipeline phenomenon? To scrutinize this, we compared the GGIs for an interest in *Science* with the GGIs for placebo interests unrelated to STEM. We found that the GGIs for placebo interests follow similar age patterns as the GGIs for the interest in *Science* across US metros. Second, we attempted to control for the time spent on the platform by computing a usage intensity gender ratio based on the difference between daily and monthly active users. This analysis showed that the usage intensity gender ratio is higher among teenagers (13–17 years) than other older age groups, suggesting that teenage girls are more engaged on the platform that teenage boys. We hypothesize that usage intensity differences, rather than inherent interest changes, might create the illusion of a leaky pipeline. Despite the previously demonstrated value and huge potential of social media advertisement data to study social phenomena, we conclude that there is little evidence that this novel data source can measure the decline in interest in STEM for young women in the USA.

## Introduction

Gender equality in Science, Technology, Engineering, and Mathematics (STEM) is vital for the achievement of the internationally agreed development goals, including the 2030 Sustainable Development Goals (SDG). Over the past decades, the global community has made concrete effort in inspiring and engaging women and girls in science. Yet women and girls continue to be excluded from participating fully in science (United Nations, 2022). On average, only around 30% of the world’s researchers are women. Moreover, less than a third of female students choose to study higher education courses in subjects like math and engineering (Wood, 2021).

The under-representation of women in STEM fields can be explained by multiple theories, including the “leaky pipeline” metaphor. The leaky pipeline, proposed in 1983, refers to the process by which initially in childhood, people of different genders show similar proportions of interest in the STEM sector. However, gender stereotypes and biases are internalized as they grow up and reach adolescence. This leads to the loss of women in STEM and arguably other fields before reaching senior roles (Berryman, 1983).

Modeling the leaky pipeline remains a challenge to apply nationally or globally. Worldwide surveys and censuses are proportionately expensive and time-consuming (Kashyap et al., 2020). Towards filling this gap, our article presents a different methodology to mirror the leaky pipeline phenomenon by examining the potential of using online sources of data, particularly data provided by the Facebook and Instagram Advertising Platforms.

Concretely, Facebook and Instagram provide their advertisers access to the number of users through their targeted advertising platform. Before an ad is launched, and before any cost is incurred, advertisers are provided with an estimate of how many users are likely to match specific criteria, which can include age, gender, sets of interests, specific locations, and much more. A recent work (Vieira & Vasconcelos, 2021) used Facebook Advertising data to assess the gender gap in STEM in Brazil. Building on this work, we focus on using Advertising data for analyzing the gender gap in STEM across age groups in the USA. The USA is an interesting case study because the majority of Americans say they use Facebook, while the use of Instagram is especially common among adults under 30 (https://www.pewresearch.org/internet/2021/04/07/social-media-use-in-2021/). The USA is also an interesting scenario because although women make up nearly half of the U.S. workforce, they are only 27% of STEM workers (https://www.census.gov/library/stories/2021/01/women-making-gains-in-stem-occupations-but-still-underrepresented.html).

In this article, we investigate the feasibility of using Facebook and Instagram advertising audience estimates for modeling the leaky pipeline phenomenon in the USA. In particular, we filter the advertising audience estimates by location, age, gender, and interests related to STEM. Then, we compute the gender gap index and compare it across age groups at the US metro level. We perform the analysis at the US metro level because a country such as the USA is very diverse and has different cultural norms in different geographical areas. Some of the metros are more conservative, while others are more liberal. Hence, looking at the USA national level might hide regional variation in gender gaps. Unlike previous work, we perform validity checks in the form of estimates of “placebo” interests and usage intensity gender ratio analysis on Instagram. To the best of our knowledge, Instagram ads audience estimates have not been rigorously tested for data quality and face validity. As such, we demonstrate the dangers of using social media advertisement data as a “black box”, and provide methodological tools for future research of such social media data novel source.

## Related work

### Gender gap in STEM

Reducing the gender gap in Science, Technology, Engineering, and Mathematics (STEM) is essential to achieve the sustainable developing goals, including Goal 5 on Gender equality. According to the United Nations, women are still excluded from participating fully despite the efforts that have been made to inspire women to study and work in STEM (Wood, 2021). Overall, women make up small percentages of STEM graduate students, tenure-track faculty, and tenured faculty (Ceci et al., 2014). Women still pursue STEM degrees at much lower rates than their male peers despite the reversal of the gender gap in educational attainment and near gender parity in math performance (Legewie & DiPrete, 2014). In most countries, a smaller percentage of women than men graduate with a degree in STEM (Card & Payne, 2021). In addition, women worldwide compromise less than 30% of STEM researchers (United Nations, 2022). Girls are less likely than boys to aspire to STEM occupations, even when they have comparable abilities and self-evaluations in math and science (Mann & DiPrete, 2016).

Research shows that women tend to gravitate away from STEM careers due to a relative abundance (*i.e*., surplus) of interests that make non-STEM careers equally or more appealing (Cardador, Damian & Wiegand, 2021; Wang & Degol, 2017). Along these lines, several studies show that women are more interested in jobs involving people and social interactions such as life sciences or health-related professions. In contrast, men are more interested in jobs involving physical objects and abstract concepts such as physics, mathematics, or engineering (Legewie & DiPrete, 2014; Ceci et al., 2014; Han, 2016; Wang & Degol, 2017; Delaney & Devereux, 2019). As such, for the past three decades, the percentage of bachelor’s degrees earned by women in biological sciences and chemistry has increased. However, the percentage of bachelor’s degrees earned by women in physics and engineering has stayed stagnant, and in other fields such as computer science, the percentage of degrees earned by women has decreased (Wang & Degol, 2017; Cowgill et al., 2021).

There are many reasons for the lower representation of women in STEM. Gender stereotypes, gender inegalitarian attitudes or discrimination against women are the main reasons that push women away from STEM fields (Soylu & Adams, 2020). Moreover, a survey of college students suggests that lack of interest, feeling unwelcome, and having concerns about the coursework diﬃculty are reasons that women differentially avoid STEM fields (Carrell, Page & West, 2010). Other research finds that lower performance of girls compared with boys in math and science at school, lack of role models (*e.g*., female teachers/professors in math and science) and self-confidence (*e.g*., the competitive environment associated with STEM careers) are factors for the lower representation of women in STEM careers (Zhou et al., 2017; Gomez, Alvarado & Nisperuza, 2020). Similar research tried to summarise six explanations for women’s under-representation in math-intensive STEM fields in the USA. These are (a) cognitive ability, (b) relative cognitive strengths, (c) occupational interests or preferences, (d) lifestyle values or work-family balance preferences, (e) field-specific ability beliefs, and (f) gender-related stereotypes and biases (Wang & Degol, 2017). Other research revealed that the under-representation of certain groups in STEM fields is not only a consequence of lower levels of academic qualifications, but also a consequence of lower levels of interest and inclination toward such fields (Riegle-Crumb & King, 2010). The research found that interest in STEM is indeed an essential factor to consider as boys often express higher self-eﬃcacy, more joy in science, and a broader interest in science than do girls (Stoet & Geary, 2018).

The under-representation of women in science, technology, engineering, and mathematics (STEM) fields can be explained by several theories, including the “leaky pipeline” metaphor. According to this metaphor, women are more likely than men to leave STEM fields at multiple time points from the beginning of college through academic tenure (Miller & Wai, 2015). In childhood, girls and boys tend to show similar proportions of interest in the STEM sector. However, this changes as people reach adolescence as gender stereotypes play a role in decreasing the number of women who are interested in STEM. This decrease happens gradually until it reaches tertiary and doctorate levels of education (Stoet & Geary, 2018). In other words, the under-representation of women in STEM, especially in computer science, engineering, and physics begins before college and is attributable to a failure to attract women to enter these fields and stay (Cheryan et al., 2017). In addition, females leave STEM fields at higher rate than do men even when females perform as well or better than their male peers on STEM-related tests or projects (Hill, Corbett & St. Rose, 2010; Reinking & Martin, 2018). Accordingly, the pipeline model sheds light on the importance of increasing the volume of flow of females from grade school to graduate school and preventing “leakage” down the line at all stages (Xu, 2008).

Several studies have evaluated and monitored the gender gap in STEM worldwide. For example, Verdugo-Castro, García-Holgado & Sánchez-Gómez (2019) reviewed instruments and questionnaires related to the gender gap that exists in the field of education within the STEM sector. The review did not only include questionnaires that dealt with the existing gender gap in the STEM sector but also examined particular matters such as the relationship between the choice of studies to be carried out, the influence of parents, self-confidence in the STEM sector, the gender gap in computer engineering, and stereotypes between men and women in adolescence. Focusing on projects funded by the European Union, García-Holgado et al. (2019) analysed several research projects about the gender gap in STEM in different calls across the last 5 years. The analysis acknowledged that most of the projects are focused on STEM or STEM subjects, STEM careers, STEM jobs or entrepreneurship, and different technologies such as robotics or IoT. In addition, Verdugo-Castro et al. (2020) designed a questionnaire to study gender stereotypes about STEM studies among university students. The study revealed that elements that influence students’ opinions are not only the contextual, family, and social ones. Factors also include educational experience and the level of education attained, prior interest in the STEM sector, previous vocational training, and branch of study chosen are all factors that shape the student’s opinion of higher STEM studies. Verdugo-Castro, García-Holgado & Sánchez-Gómez (2020) conducted interviews with Spanish women working in STEM. Some of the interviewees admitted that they were encouraged to take STEM and try it out, while others admitted that they were afraid to pursue STEM studies. Similarly, García-Holgado, Díaz & García-Peñalvo (2019) interviewed women as a part of the W-STEM project, which seeks to improve strategies and mechanisms for attracting, accessing, and guiding women in Latin America in STEM higher education programs. These interviews were recorded and shared *via* different channels to make the profiles of these STEM role models visible.

Researchers have offered guidelines and suggestions to attract women to study and work in STEM fields. In particular, Tobar Subía Contento & Nohemi Gamez Aparicio (2020) recommended holding attraction campaigns where the female role models in STEM careers are highlighted, as well as providing data on the number of women studying STEM. Furthermore, the study recommended organizing talks for students to promote vocations in STEM areas. Other studies such as Bennaceur et al. (2018) proposed creating programs and science festivals that encourage girls to study engineering from an early age. The study also proposed developing mentoring programs and promoting the visibility of women as role models through diverse social media channels such as YouTube, Facebook, and Twitter. González-González & García-Holgado (2021) agreed that it is essential to start STEM programs with students at a young age and show them that girls and boys can study and work in STEM. To promote role models in the domain of computer science and software engineering, Ribaupierre et al. (2018) advocated in favour of having a good ratio of women and men on the university teaching team and in favour of good ratio of women and men by software engineering and computer science sub-area. Other work such as Torres-Ramos et al. (2020) stressed the importance of “self-eﬃcacy,” known as the ability to accomplish a given task to attract more women and girls in STEM fields. Encouraging and supporting girls’ self-eﬃcacy can be achieved by “academic advising, faculty mentorship, tutoring, internship opportunities, and career and skill development” (ibid.). After finding that girls reported significantly lower levels of technical self-eﬃcacy and lower interest in computer science than boys, Brauner et al. (2018) offered several guidelines to adjust children’s mental model, including inviting guests from STEM disciplines into the classrooms, organizing Girls’ Days, inviting boys and girls into universities for research internships and launching events that help boys and girls understand computing principles and increase their technical self-eﬃcacy.

Quantitative and qualitative techniques have been implemented previously to address the gender gap in STEM. For example, a survey has been used to explore patterns of individuals talented in STEM and determine whether these patterns and experiences differed for men and women or women from different age groups (Heilbronner, 2013). Furthermore, a survey research method was followed to assess the situation of women in STEM in Latin America and Europe and observe if there is any significant gender gap (García-Holgado et al., 2020). Focusing on primary school students, a questionnaire has been designed to study if there are gender differences in aspects related to the experience with mathematics (Ayuso et al., 2021). Focusing on undergraduate students, a survey has been distributed to look at the preponderance of female and male students in science-oriented majors leading to STEM careers connected to internal and institutional factors (Faitar & Faitar, 2013). In addition, a survey was carried out to understand the perception of students/teachers and analyze the factors of the gender gap in STEM (Moreno et al., 2014). Other studies relied on existing survey data to study topics related to the gender gap in STEM. For instance, the Freshman Survey data collected by the Cooperative Institutional Research Program (CIRP) was used to analyse how the characteristics of women planning to majors vary across different STEM subfields (Sax & Newhouse, 2018). The Engineers Statistical Data System (SESTAT) was also used to assess the factors influencing the gender gap in persistence in STEM employment in computer science and engineering occupations (Sassler, Michelmore & Smith, 2017). The Educational Longitudinal Surveys of 2002 to 2012 (ELS) was used to assess the gender gap in STEM major completion (Weeden, Gelbgiser & Morgan, 2020), while the 2006 PISA dataset was used to study the gender gap in STEM career expectations across countries (McDaniel, 2016). Other researchers used the NCES (National Center for Education Statistics) longitudinal surveys to consider whether males see their life goals as more compatible with STEM careers than females (Mann & DiPrete, 2013). Other studies applied pre-post surveys to evaluate different kinds of programs that aim to increase females’ interest in and enthusiasm for science through (Levine et al., 2015; Levine & DiScenza, 2018). Other researchers conducted interviews with women in STEM professions to capture the low participation of women in STEM professions along with the challenges they face in their working environment (Tandrayen-Ragoobur & Gokulsing, 2021).

### Facebook Ads data

Before showing Ads to Facebook users, Facebook allows advertisers to estimate the potential audience estimate, *i.e*., estimates of how many people an ad could potentially reach, given specific criteria and targeting options. For example, the monthly potential reach estimate for a Facebook-proper audience, not counting Instagram or Messenger, located in the USA, aged 13 to 50+, and female is 67,000,000. The potential reach audience estimate is available for free in the Facebook Ads Manager (https://www.facebook.com/business/help/200000840044554?id=802745156580214). The estimated audience size can be filtered by age, gender, location, and device type. In addition, the estimated audience size can be filtered by users’ interests. The interests filter depends on what Facebook users engage with and like on the platform. Facebook determines a user’s interests based on the pages they like, the content they view, and the ads they click (https://www.facebook.com/help/562973647153813).

Facebook’s audience estimates have been used for a range of studies connecting society and the world’s online population. For example, researchers took advantage of Facebook’s audience estimate to predict gender gap in internet use (Kashyap et al., 2020). The monthly active Facebook users have been filtered by age, gender, and country. The study demonstrated that advertising digital data from Facebook can be used to complement traditional data sources to monitor global development indicators linked to digital gender inequality. Similar research worked on filtering Facebook Ads data by gender, geography, age, education, industries, and device type in order to examine variation in gender gaps in India (Mejova et al., 2018). The study found that there is a significant sub-national variation in gender gaps in Facebook use and higher gender gaps tend to be present in states with greater gender inequality in schooling and lower levels of social and economic development. Other researchers developed a metric called Facebook Gender Divide (FGD) to measure gender differences in Facebook access and activity in 217 countries (Garcia et al., 2018). The FGD tends to be associated with other types of gender inequality, including economic, health, and education inequality. Recently, researchers have looked into using Facebook Ads data to measure the gender gap in mobile device usage duration and saw patterns of gender gaps disfavouring women, especially among younger people (Sabri, Kashyap & Weber, 2021). In a different article, Gil-Clavel & Zagheni (2019) extended the scope and used Facebook Ads data to include the role of age. The authors described that in countries in North America and Northern Europe, patterns of Facebook adoption do not differ significantly between older and younger adults. In Asian countries, which have high levels of gender inequality, differences in levels of Facebook adoption by gender disappeared at older ages (ibid.).

Facebook Ads data has also been leveraged to study culture and assimilation. In a recent study, Facebook data was filtered by geography and interests related to specific cultural traits, providing a collection of massive data on human behaviors that are used to complement traditional cultural metrics (Obradovich et al., 2020). Moreover, Facebook Ads data has been filtered by geography and interest in Brazilian dishes in order to focus on how a foreign culture such as the Brazilian culture is spread around the world (Vieira et al., 2020). In parallel, researchers found that Facebook Ads data can be used to explore the cultural assimilation of Mexican Immigrants by looking at musical preferences. Accordingly, Facebook Ads data has been filtered by interest in musical genres and Facebook’s ethnic aﬃnities demographic attributes (Stewart et al., 2019). Also, researchers disclosed that Facebook data can provide insights into the assimilation of Arabic-speaking migrants in Germany. For this, Facebook data has been collected and filtered by interests and by different populations (*e.g*., non-expats living in Germany, or Arabic-speaking expats living in Germany) (Dubois et al., 2018).

Facebook Ads data has been used to reinforce research in the political science and health science domains. For instance, the research looked into leveraging Facebook Ads data to infer the audience demographics of politicians in the electoral race (Ribeiro, Kansaon & Benevenuto, 2019). Here, Facebook Ads data has been filtered by interests related to candidates to calculate audience demographics. In the context of health, Facebook Ads data has been collected to compute the proportion of users in a target demographic group with interest related to schizophrenia (Saha et al., 2017). This data has been used to construct a plausible index of population-scale schizophrenia awareness. Furthermore, Facebook Ads data has been utilized to track health conditions associated with tobacco use, obesity, and diabetes (Araujo et al., 2017). This research contributes to previous work by performing quality check in the form of comparing the performance of placebo interest. In consideration of that, Facebook Ads data has been collected and filtered by country, interests in health conditions, interest in placebo, age, and gender. Equivalently, previous work introduced the use of placebo interests and compared these interests to interests related to four diverse health conditions: (1) diabetes (type II), (2) obesity, (3) food sensitivities, and (4) alcoholism (Mejova, Weber & Fernandez-Luque, 2018). The study showed that placebo interest such as Reading and Technology display a nontrivial correlation with other health interest such as Diabetes and Obesity. This makes measuring placebo interests a critical step in verifying the significance of health-specific results.

Assessing the gender gap in STEM has been scrutinized using Facebook Ads data. Focusing on Brazil as a case study, Vieira & Vasconcelos (2021) argued that even with a larger proportion of women on Facebook, STEM interests are still concentrated towards men. In particular, Facebook Ads data disclosed that college majors related to Environmental Science, Engineering, and Computer Science have more men interested in them. However, college majors related to Life Science and Math/Physical are preferred by women (ibid.). Additionally, Vieira & Vasconcelos (2021) (ibid.) applied gender balance analysis on demographic subgroups, in particular for education levels and age groups. The analysis confirmed that the gender gap in STEM widens significantly in the transition from Bachelor’s to post-graduate levels (*e.g.*, Masters or Doctorate levels) as well as into research careers. The authors also compared Facebook users’ age with their college major interests. Explicitly, the authors split the population into four subgroups: adolescent (13–19 years), early adulthood (20–39 years), adulthood (40–64 years), and maturity (65 years or more). The authors observed the same pattern as the educational level where the proportion of female Facebook users interested in each major decreases as older as they are. Over their lifetime, women tend to lose even more interest in STEM majors such as Aviation and Mechanical Engineering.

### LinkedIn ads data

Similar to Facebook, LinkedIn allows advertisers to estimate the reach of their campaigns. For instance, in 2017 LinkedIn estimated that an advertisement “targeted to 18- to 24-year-old males in San Francisco Bay Area with knowledge in Java, has the potential to reach 11,000 people” (Haranko et al., 2018). Such estimates of employment and skills are hard to obtain using other traditional data sources. LinkedIn advertisement data has been filtered by skills, industry, age, location, and gender in order to understand gender gaps in employment along various dimensions (ibid.). The study reported that Education and and Medical/Health care seem to be the most female dominant categories, whereas Construction and Manufacturing seems to be the most male dominant categories. The study also reported that there is a high correlation between LinkedIn data and traditional data source provided by the US Bureau of Labor Statistics. Building on this work, LinkedIn data has been disaggregated by job seniority, job function, field of study, industry, age, location, and gender to examine gender inequalities in the professional domain (Kashyap & Verkroost, 2021). This research outlined that gender inequality on LinkedIn is exceptionally high among older individuals and in Africa, the Middle East, and parts of Asia. The study agrees that LinkedIn data is correlated with traditional data sources available from the International Labour Organization’s (ILO) Statistical Database. Furthermore, LinkedIn data has been disaggregated by industry, location, and gender to examine variation in the professional gender gap in IT industry (Verkroost et al., 2020). The authors observed that in the large majority in countries worldwide, there are more men than women in IT. Specifically, there are almost four times more men than women in IT industries like networking and computer hardware, while there are almost twice as many men as women in the telecommunication and internet industries. The authors also observed that LinkedIn data tend to be similar to other traditional data sources available from Eurostat, at least for European countries.

The previous studies discussed above use LinkedIn and Facebook Ads data to monitor digital and employment gender gaps, culture, politics, health, and wellbeing. Our study is instead focused on the combination of Facebook and Instagram data for measuring gender gap in STEM. Different from research that explores the gender gap in STEM in Brazil, this research examines the gender gap in STEM in the USA. Specifically, our study is different from Vieira & Vasconcelos (2021)’s work in that it considers different geography, platforms, and age groups. We aim to focus on the gender gap in STEM among teenagers and young adults. Our work builds on previous research strands by comparing gender gap in advertisement data across age and USA metropolitan area. Additionally, our research introduces measuring placebo interests and usage intensity gender ratio in order to validate findings related to gender gap in STEM. This is an important methodological contribution as it shows that many apparent differences in terms of interest profiles might be explained by differences in time-spent-on-platform.

## Methodology

### Data collection

**Figure 1:**
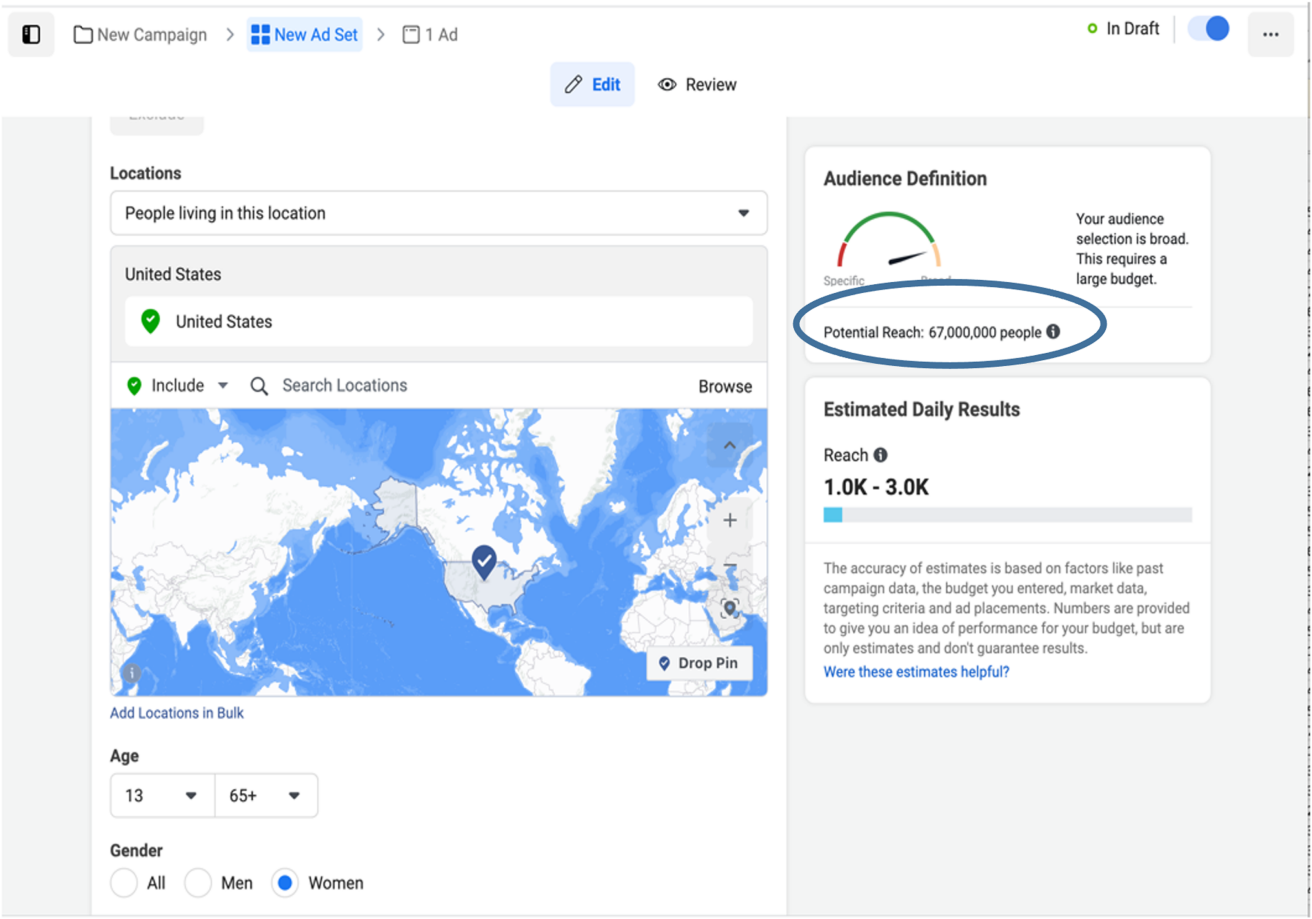
Facebook Ads Manager.

Facebook and Instagram data has been retrieved from Facebook’s Marketing application programming interface (API). The Web interface of the Ads Manager (https://www.facebook.com/business/tools/ads-manager) can render equivalent data, but using the API makes programmatic access easier (Mejova, Weber & Fernandez-Luque, 2018). Figure 1 shows a screenshot of the Facebook Ads Manager illustrating the potential reach to an audience likely to match targeting criteria. The Facebook marketing API provides two metrics: Daily Active Users (DAUs) and Monthly Active Users (MAUs). On Facebook for developers, DAUs are defined as the “estimated number of people that have been active on your selected platforms and satisfy your targeting spec in the past day” (https://developers.facebook.com/docs/marketing-api/reference/ad-campaign-delivery-estimate/), while MAUs are defined as the “estimated number of people that have been active on your selected platforms and satisfy your targeting spec in the past month” (https://developers.facebook.com/docs/marketing-api/reference/ad-campaign-delivery-estimate/).

The DAUs and MAUs reach estimates have been queried from the API and filtered by location, age, gender, interest, and platform as the following:

**Location**: This covers all the USA metropolitan statistical areas (https://en.wikipedia.org/wiki/Metropolitan\_statistical\_area) (210 metros). We collected geographical data to capture variations in gender gaps in the USA.

**Age**: This covers various age groups (13–16, 14–17, 15–18, …26–29 years old) as well as all age groups (13–29 years old).

**Gender**: This covers female, male, and all genders.

**Interest**: We used the Facebook Ads Manager interface to identify the key interests related to STEM. As such, we filtered the reach estimate by four interests: *Science, Technology, Engineering*, and *Mathematics*.

**Platform**: This covers two platforms: Facebook (this includes all platforms such as Facebook, Instagram, Audience Network and Messenger), and Instagram-only.

We included Instagram-only as Instagram is known to be popular with teenagers (https://www.statista.com/statistics/248769/age-distribution-of-worldwide-instagram-users/.). The MAUs metric does not report numbers under 1,000 to prevent the targeting of small groups of individuals. We are including MAUs values under 1,000 in the analysis, but we are replacing these small values with NAs (On Facebook, we got 3,881 NAs out of 12,600 data points. On Instagram, we got 4,966 NAs out of 12,600 data points).

We also collected the number of users not filtered by any interest from Facebook and Instagram, in order to calculate the Gender Gap index.

### Gender gap index

We have used the MAUs to calculate the Gender Gap Index (GGI) for each USA metro and age group as the following:

(1) $${\rm GGI = }\displaystyle{{{\rm Female\; to \;male \;gender\; ratio\; filtered\; by \;each\; STEM \;interest}} \over {{\rm Female \;to\;male\; gender\; ratio \;of\; the\; number\; of\; users\; in \;each \;platform}}}$$

A GGI less than 1.0 indicates a gender gap with women at a disadvantage. Conversely, a value equal to or larger than 1.0 indicates that gender parity for the interests considered has been achieved or exceeded.

## Results

### Min and max GGIs

In this section, we report the min (= worst for women) and max (= best for women) GGI values for age groups 13–16 and 26–29 years on Facebook and Instagram (interest in *Science*). On Facebook, the min GGI for age group 13–16 years belongs to Harlingen-Wslco-Brnsvl-Mca (0.982), while the max GGI for the same age group belongs to Orlando-Daytona Bch-Melbrn (1.516). For age group 26–29 years, the min GGI belongs to Laredo (0.885), while the max GGI for the same age group belongs to Greenwood-Greenville (1.49). On Instagram, the min GGI for age group 13–16 years belongs to Salt Lake City (1.095), while the max GGI for the same age group belongs to Dallas-Fort Worth (1.642). For age group 26–29 years, the min GGI belongs to El Paso-Las Cruces (1), while the max GGI for the same age group belongs to Columbus-Tupelo-West Point-Houston (1.725). Table 1 shows the top and bottom three GGI values for age group 13–16 years old, while Table 2 shows the top and bottom three GGI Values for age group 26–29 years old. We expected to see Metros with low GGI (<1), especially with small Metros that might have higher gender gaps. However, this is not the case as there are many GGI values that are >1 in Facebook as well as Instagram (On Facebook, we obtained 4,092 GGI values that are <1, while we obtained 4,627 GGI values that are ≥1 out of 12,600 data points. On Instagram, we obtained 2,862 GGI values that are <1, while we obtained 4,772 GGI values that are ≥1 out of 12,600 data points. These GGIs include interests related to *Science, Technology, Engineering*, and *Mathematics*).

**Table 1:** Top and bottom three GGI values for age group 13–16 on Facebook and Instagram (interest in Science).

Platform	Top metros	Bottom metros
Facebook	Birmingham (Ann and Tusc) (1.4)	Harlingen-Wslco-Brnsvl-Mca (0.982)
	Miami-Ft. Lauderdale (1.438)	Fresno-Visalia (1.006)
	Orlando-Daytona Bch-Melbrn (1.516)	San Diego (1.01)
Instagram	Chicago (1.611)	Salt Lake City (1.095)
	Seattle-Tacoma (1.626)	Minneapolis-St. Paul (1.307)
	Dallas-Ft. Worth (1.642)	San Francisco-Oak-San Jose (1.339)

**Table 2:** Top and bottom three GGI values for age group 26–29 on Facebook and Instagram (interest in science).

Platform	Top metros	Bottom metros
Facebook	Duluth-Superior (1.467)	Laredo (0.885)
	Columbus-Tupelo-W Pnt-Hstn (1.477)	San Angelo (0.936)
	Greenwood-Greenville (1.49)	El Paso (Las Cruces) (0.939)
Instagram	Columbia-Jefferson City (1.608)	El Paso (Las Cruces) (1)
	Monroe-El Dorado (1.701)	Monterey-Salinas (1)
	Columbus-Tupelo-W Pnt-Hstn (1.725)	Utica (1.019)

### Comparison between GGIs across age groups

**Figure 2:**
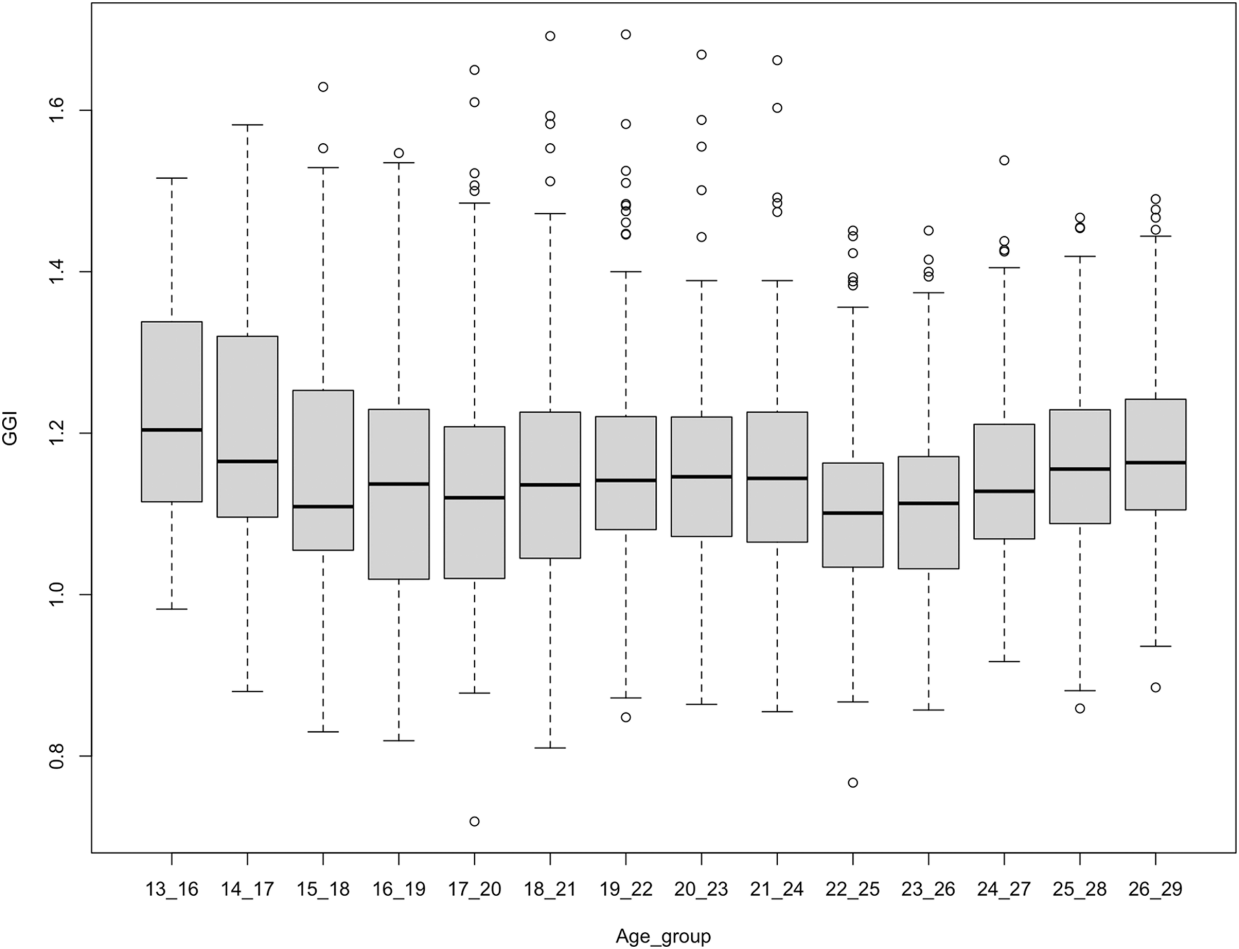
GGI for interest in science across age groups on Facebook. Each point corresponds to a different US metro.

**Figure 3:**
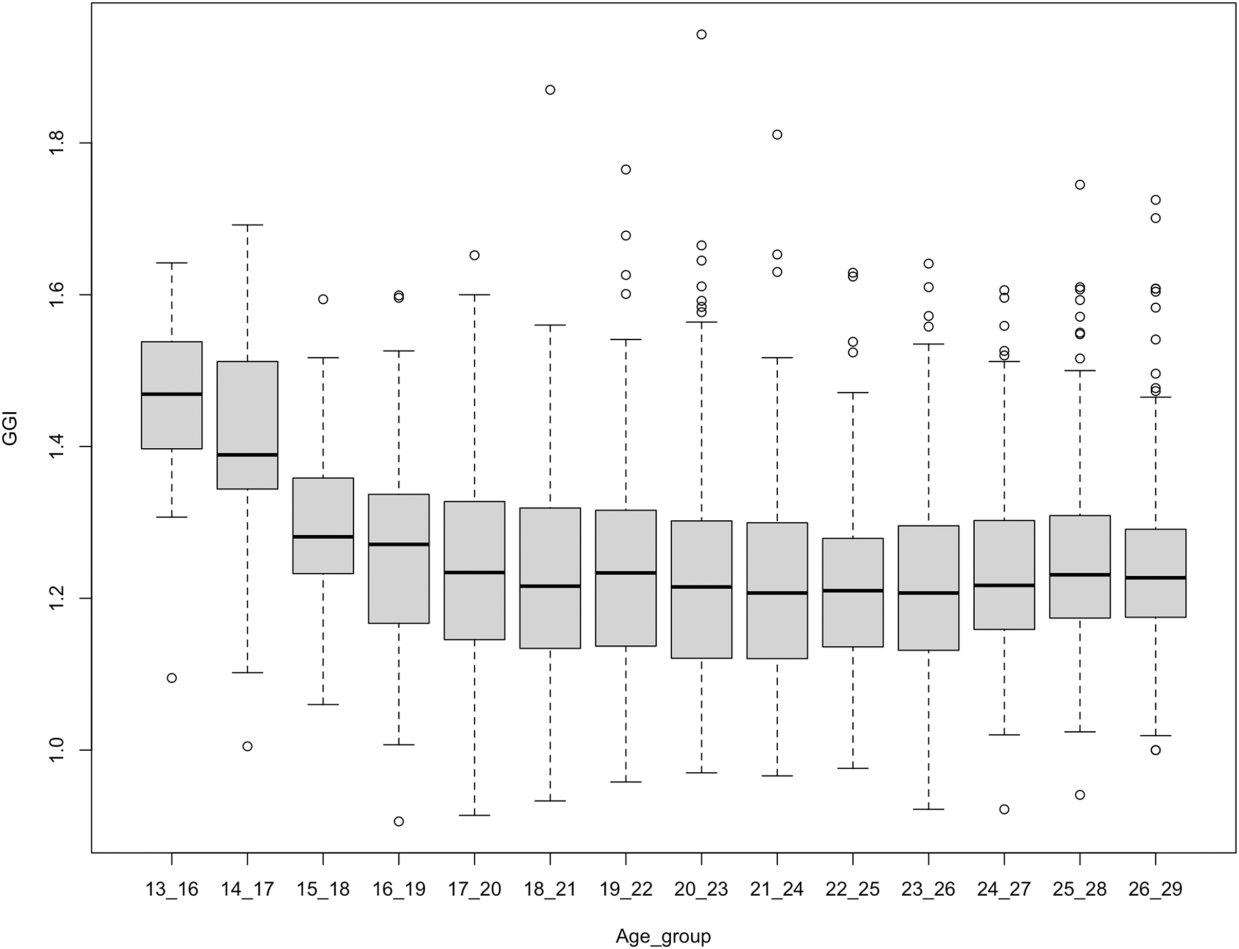
GGI for interest in science across age groups on Instagram. Each point corresponds to a different US metro.

**Figure A1:**
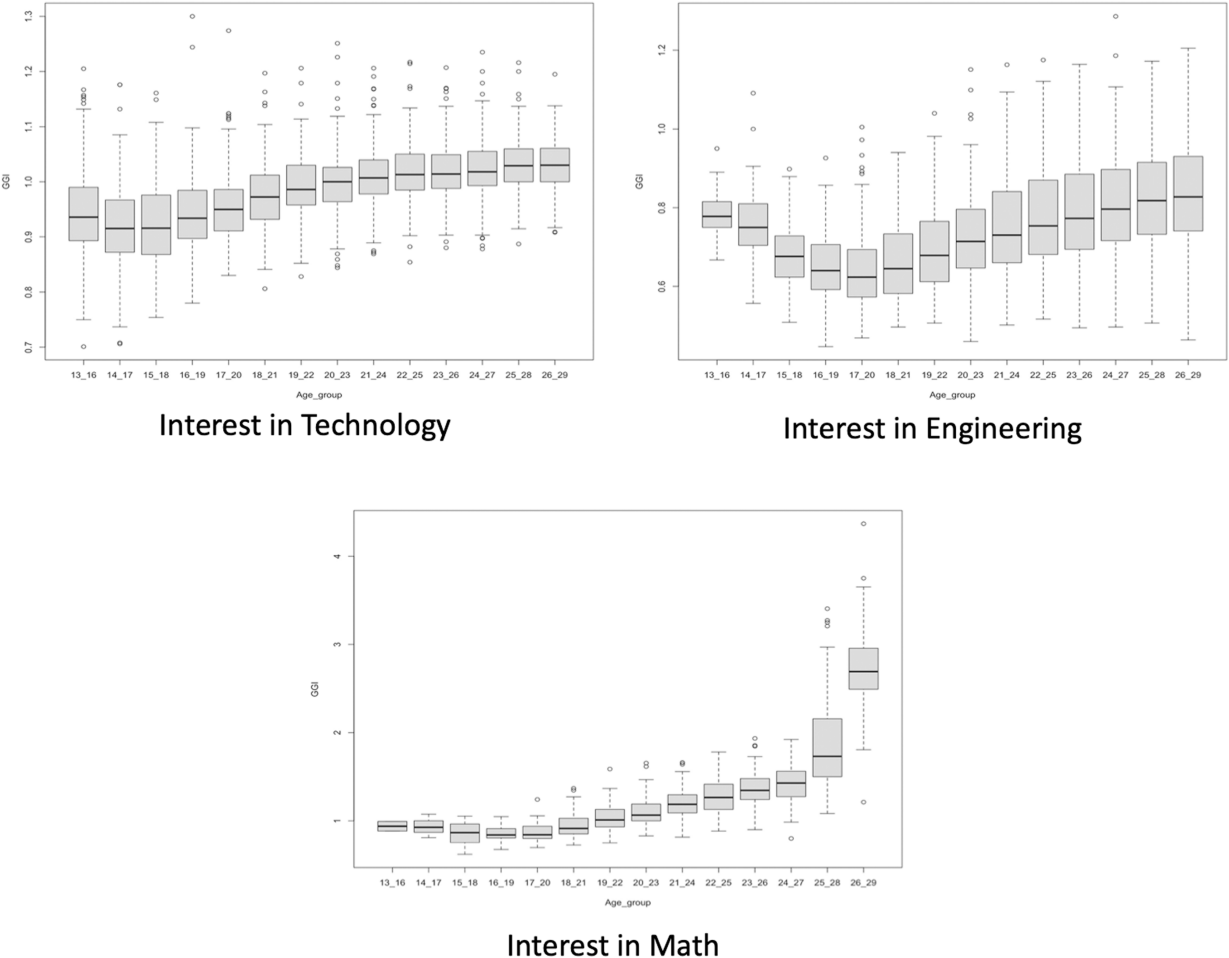
Facebook GGIs for interests in ‘Technology’, ‘Engineering’, and ‘Math’.

**Figure A2:**
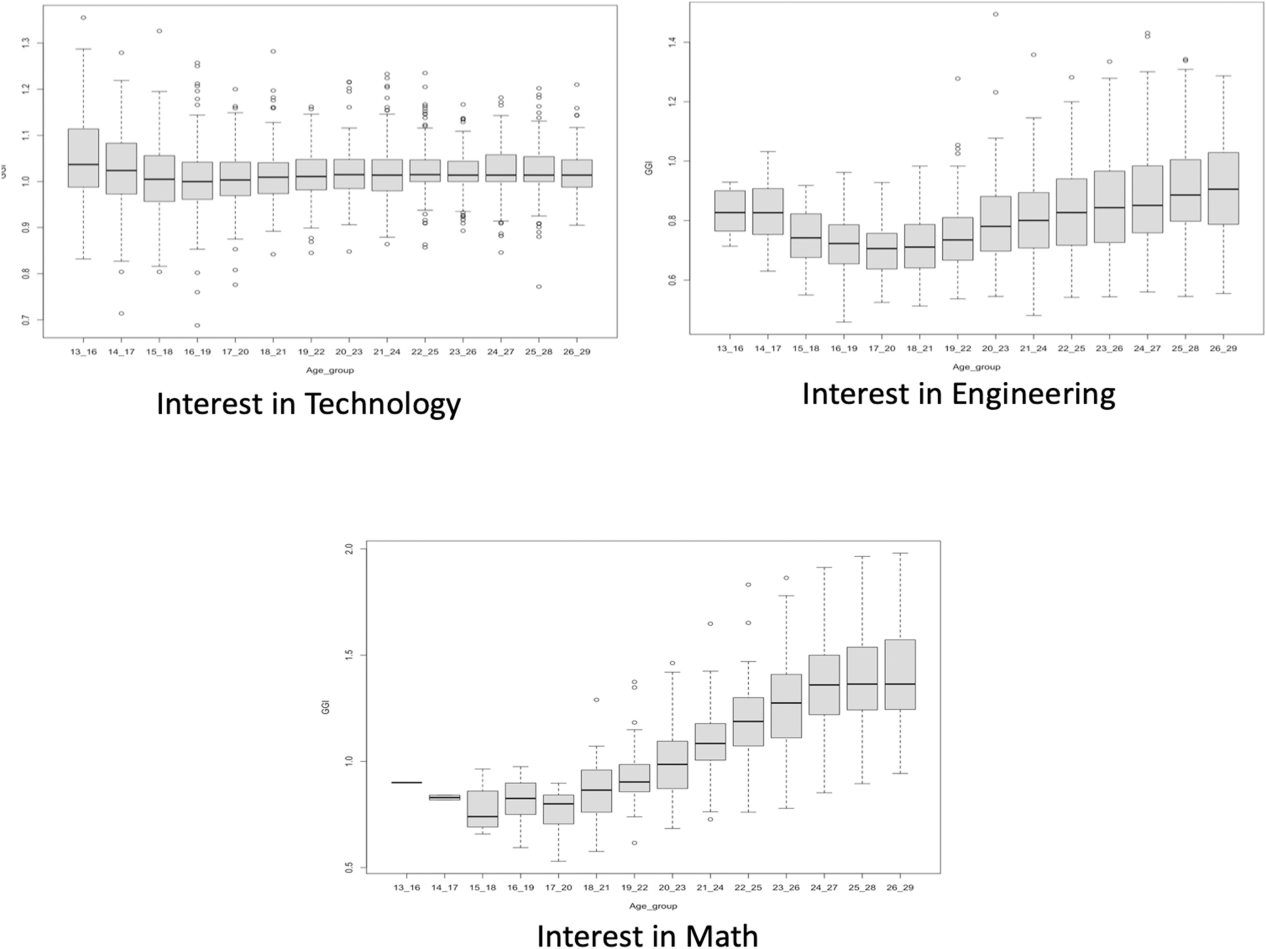
Instagram GGIs for interests in ‘Technology’, ‘Engineering’, and ‘Math’.

Here, we compare the GGI across age groups on each platform. On Facebook, we found that the GGI of interest related to *Science* fluctuates across age groups (Fig. 2). Each point on the boxplot corresponds to a different USA metro. The GGI decreases from age group 13–16 to age group 15–18, then increases from age group 16–19 to age group 21–24. The GGI decreases again at age group 22–25 and then increases at age group 23–26 until reaching age group 26–29 years. We have conducted two tests: the Kruskal-Wallis and the Paired t-test. The Kruskal-Wallis is conducted in order to determine if there is a statistically significant difference between the GGIs for different age groups. The paired t-test is conducted in order to determine if there is a difference between the GGIs at age groups 13–16 and 26–29. After conducting Kruskal-Wallis on Facebook data, we reject the null hypothesis and conclude that not all the group medians are equal (Kruskal-Wallis chi-squared = 85.425, df = 13, *p*-value = 1.041e−12, *n* = 2,940, effsiz = 0.0248). The paired t-test results designates that there is significant evidence of change in GGIs between age groups 13–16 and 26–29 years old (t = −5.0328, df = 35, *p*value = 1.453e−05, 95% CI [−0.15179885 to −0.06453449], mean of the differences = −0.108, *n* = 210, effsiz = 0.839). The mean of the difference in GGI between the two age groups is reduced by 0.11 points. Concerning other interests on Facebook, such as interest in *Technology, Engineering*, and *Mathematics*, we did not observe a decrease in the GGI as users get older (Fig. A1). On Instagram, however, we noticed that the GGI of interest related to *Science* is decreasing across age groups, suggesting that women, compared to men, are relatively losing interest in STEM at older ages (Fig. 3). There was a statistically significant differences between GGI in different age groups as assessed using the Kruskal-Wallis test (Kruskal-Wallis chi-squared = 127.47, df = 13, *p*-value = 6.67368e−21, *n* = 2,940, effsiz = 0.0391). The carried out Paired t-test reveals that the mean paired difference between between age groups 13–16 and 26–29 years does not equal zero (t = −8.2268, df = 17, *p*value = 2.489e−07, 95% CI [−0.3296102 to −0.1950564], mean of the differences = −0.262, *n* = 210, effsiz = 1.94). The GGI between the two age groups is reduced by 0.26 points. Concerning interests on Instagram related to *Technology, Engineering*, and *Mathematics*, we did not notice a significant decrease in GGI across ages, as shown in (Fig. A2). Overall, the results denote that the GGIs on Instagram for interest in *Science* only mirror the leaky pipeline as women are losing interest in *Science* as they grow up. Specifically, (i) the relative trend across the ages is in line with a leaky pipeline, but (ii) the absolute level (actual GGI values remains > 1.0 all the time) is not in line with a leaky pipeline. Before confirming this outcome and trying to understand the trend on the relative level, we collect and analyse data related to *placebo* interests on Instagram.

### Placebo interests

Figure 3 provides potential evidence for a leaky pipeline on Instagram as the GGI is decreasing for older age groups, indicating a relative loss in *Science* of women compared to men. However, to rule out certain confounding factors, we also collected data and performed an analysis for a set of placebo interests. Placebo interests are interests on Instagram that should not have an obvious causal link with interests in STEM but that might still turn out to be correlated due to latent factors such as general engagement on the platform (Mejova, Weber & Fernandez-Luque, 2018). Placebo interests are helpful to understand if we are really measuring interest in science on Instagram and not the level of activity on the platform or other unknown latent factors. Intuitively, “these interests are meant as a placebo wherein no topic-specific treatment is performed, and any effect observed is due to the random or causal factors outside the topic” (ibid.). Similar to previous work (ibid.), we used popular generic interests that are not *a priori* linked to the four STEM interest studied on Instagram. The generic interests include *Facebook, Reading, Music*, and *Entertainment*. We also added other gendered interests such as *Cars* and *Beauty*. As many of these Placebo interests are very general with audiences of millions of users worldwide, they can be seen as a kind of proxy for general engagement on the platform: if a user really shows no sign of interest in, say, any type of music, then that could be plausible due to a lack of engagement in activities on the platform. We collected the reach estimates from the API filtered by location, age, gender, and placebo interest as the following:

**Location**: This covers all the USA metropolitan statistical areas (210 metros).

**Age**: This covers various age groups (13−16, 14−17, 15−18, …26−29 years old).

**Gender**: This covers female, male, and all genders.

**Interest**: We used the Facebook Ads Manager interface to exhaustively enumerate Placebo interests on Instagram. Then, we filtered the reach estimates by interest in *Facebook, Reading, Music, Entertainment, Cars* and, *Beauty*.

The MAUs have been used to calculate the gender gap index (GGI) for each USA Metro and age group as the following:

(2) $${\rm Placebo \;GGI = }\displaystyle{{{\rm Female\; to\; male\; gender \;ratio\; filtered\; by \;each \;placebo\; interest}} \over {{\rm Female\; to\; male\; gender \;ratio\; of\; the\; number \;of \;users\; in \;each\; platform}}}$$

**Figure 4:**
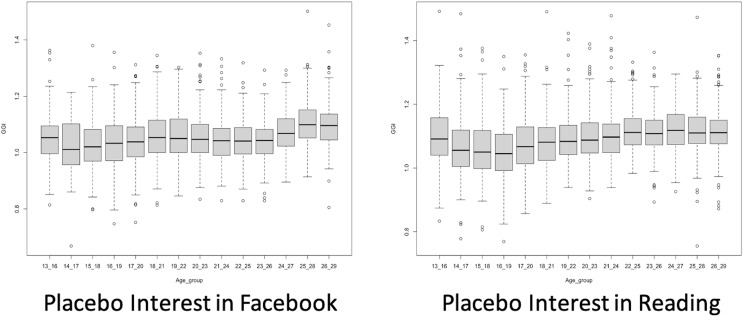
Placebo GGIs for interests in ‘Facebook’ and ‘Reading’. Each point corresponds to a different US metro.

**Figure 5:**
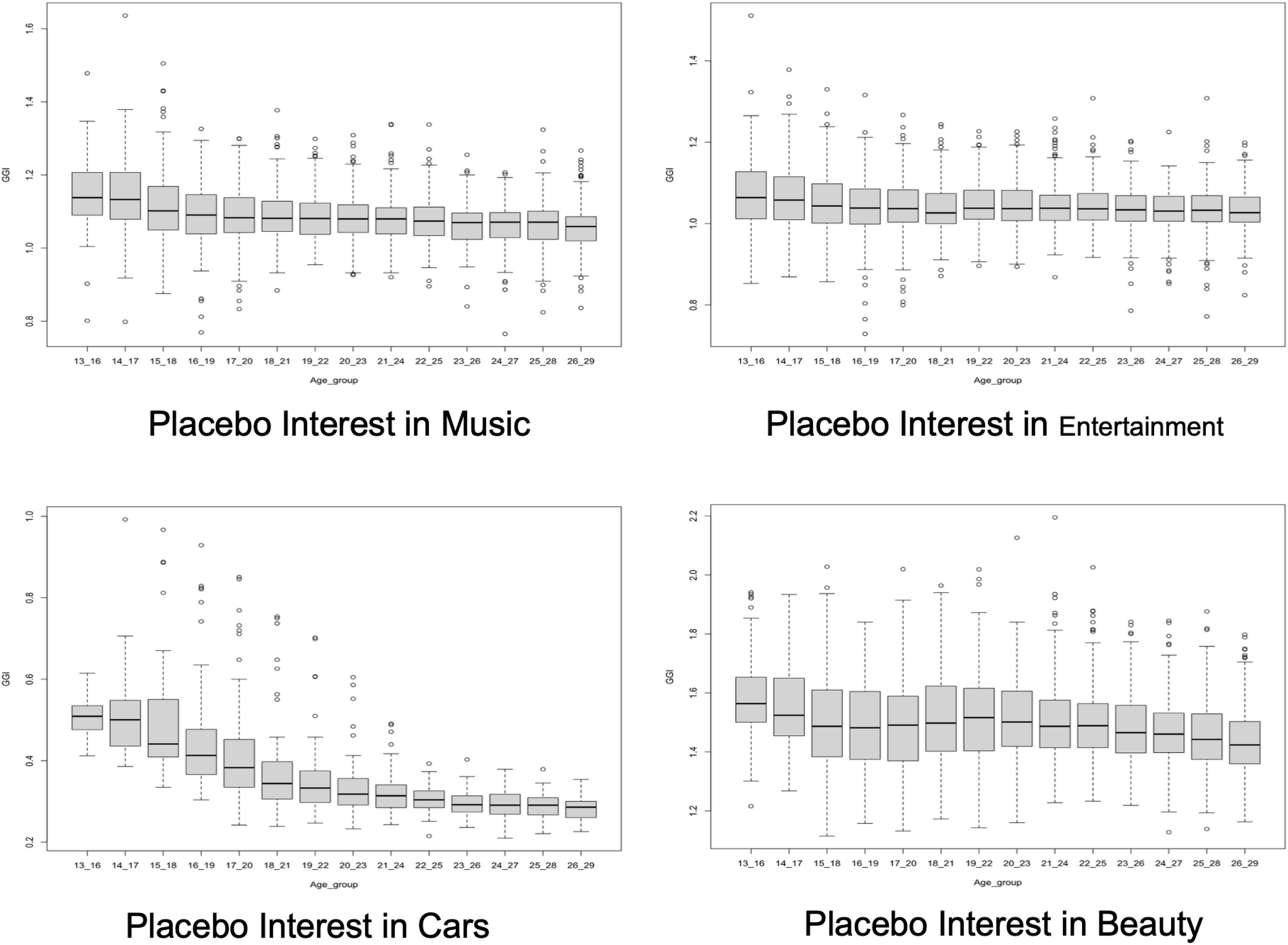
Placebo GGIs for interests in ‘Music’, ‘Entertainment’, ‘Cars’ and ‘Beauty’. Each point corresponds to a different US metro.

**Figure A3:**
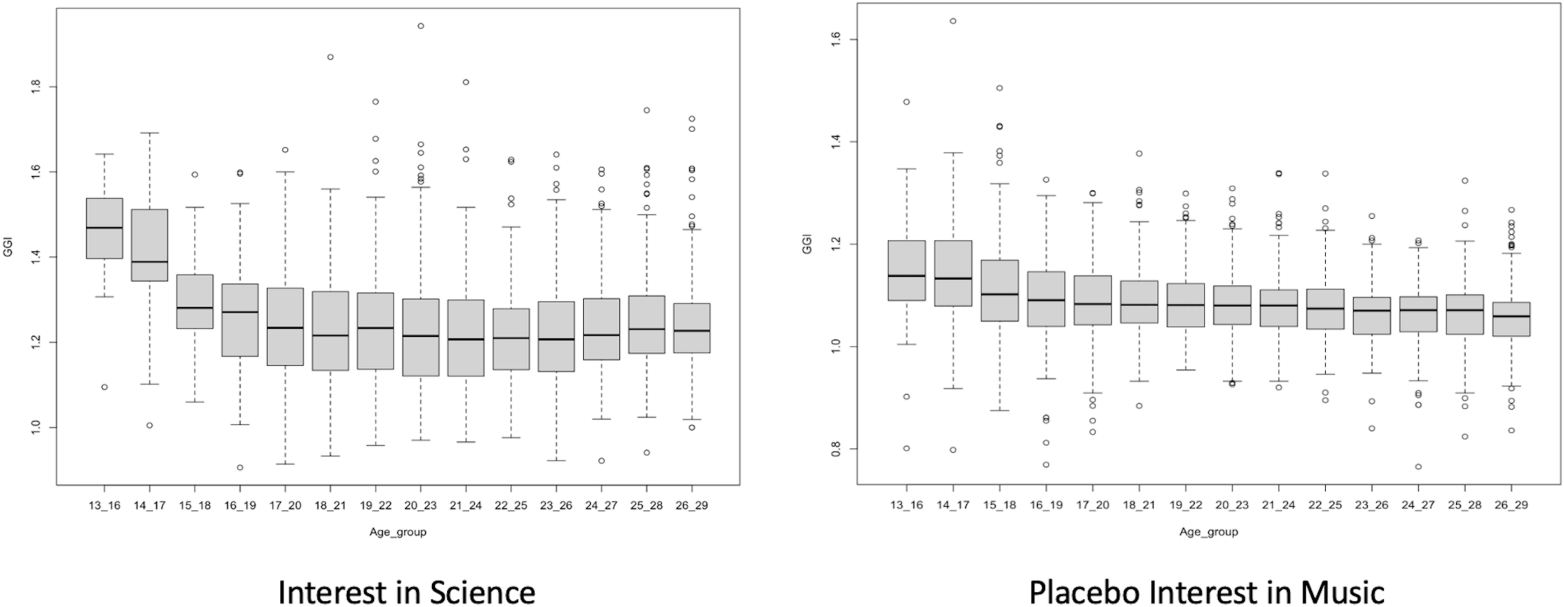
Side-by-side comparison between the GGIs for interest in ‘Science’ and GGIs for interest in placebo (music) on Instagram.

**Figure A4:**
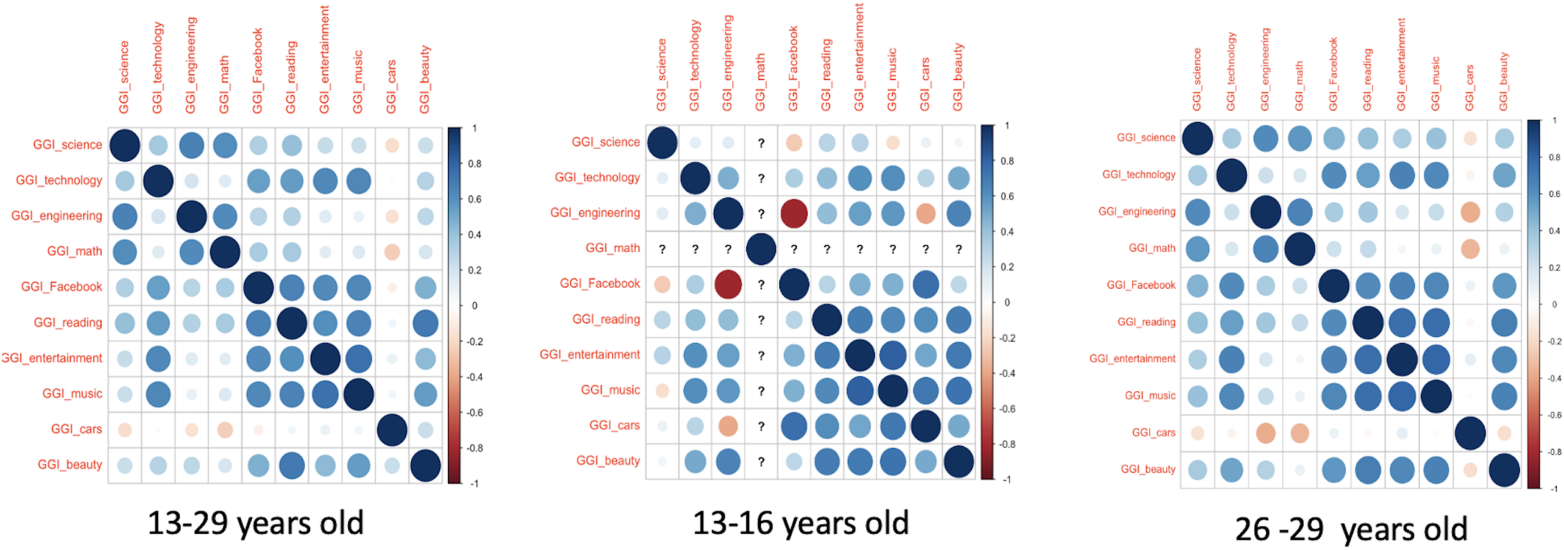
Correlation between the STEM GGIs and the placebo GGIs for age groups 13–29, 13–16, and 26–29 years old.

We discern that the placebo GGIs for interest related to *Facebook* and *Reading* increase as users get older, demonstrating that women, compared to men, are not losing interest in *Facebook* and *Reading* at older ages (Fig. 4). Nevertheless, we discern the placebo GGIs for interest related to *Music, Entertainment, Cars* and, *Beauty* decrease as users get older, showing that women, compared to men, are losing interest in *Music, Entertainment, Cars* and *Beauty* at older ages (Fig. 5). This makes the relative GGIs patterns that we observed on Instagram for interest in *Science* not unique as placebo interest follow similar patterns across age groups. For example, Fig. A3 shows that the GGIs for interest in *Science* for different age groups on Instagram have a similar pattern as the GGIs for interest in *Music*. In fact, we also observed that the 26–29 age group GGI for interest in *Science* is correlated with the GGI for interest in Facebook (*r* = 0.46), interest in Reading (*r* = 0.41), and interest in Music (*r* = 0.40). In addition, we observed that the 13–29 age group GGI for interest in *Science* is correlated with the GGI for interest in Facebook (*r* = 0.33), interest in Reading (*r* = 0.41), and interest in Music (*r* = 0.23). Within placebo interests, we observed that the 13–29 age group GGI for interest in Facebook is correlated with the GGI for interest in Reading (*r* = 0.66), interest in Entertainment (*r* = 0.63), and interest in Music (*r* = 0.63). In general, as shown in Fig. A4, the GGIs for interest in STEM (with the exception of interest in *Math*) are correlated with the GGIs for interest in placebo. As such, we cannot conclude that online data provided by Instagram reflect the leaky pipeline phenomenon when we measure GGI for interest on *Science*.

### Activity level on the platform

In the previous section, we found that the GGIs for age groups 13–16 and 14–17 years are higher than the GGIs for the other older age groups when we consider interests in *Science* as well as interests in placebo on Instagram. This difference in the GGIs could be attributed to 13–17 years old users spending more time on the platforms. Unfortunately, time spent on the platforms or usage intensity is not given by Facebook or Instagram by default. So in an attempt to control the time spent on the platform, we look at the DAUs to MAUs ratio on Instagram as:

(3) $$\eqalign{ {\rm Usage\; Intensity\; Gender \;Ratio = } \quad \displaystyle{{{\rm DAUs\; female\; to \;male\; gender \;ratio \;of \;the\; number\; of\; users\; on\; Instagram}} \over {{\rm MAUs\; female\; to\; male\; gender\; ratio\; of\; the\; number\; of\; users\; on\; Instagram}}} }$$

**Figure 6:**
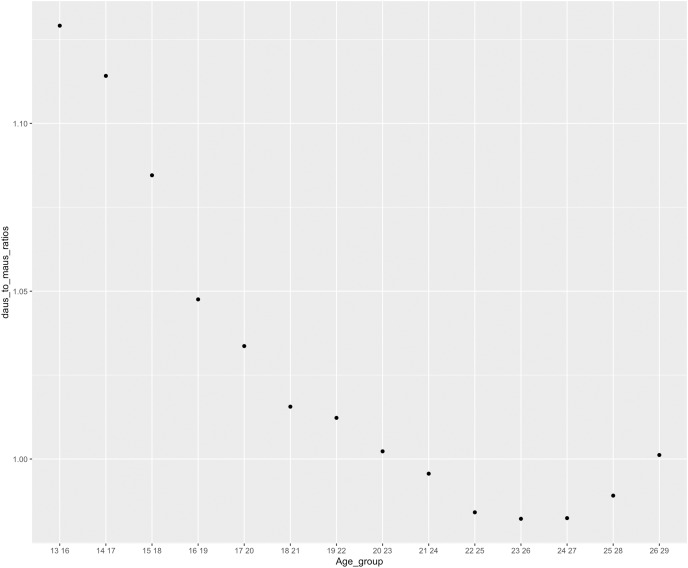
Usage intensity gender ratio across age groups (US national level).

In other words, DAUs to MAUs ratio serves as a proxy for the Usage Intensity Gender Ratio. Equation (3) captures female usage intensity divided by male usage intensity. For user groups who spend more time on the platform, we would expect a bigger proportion of the monthly active users (MAUs) also to be daily active users (DAUs). Correspondingly, we expect a higher DAUs to MAUs ratio and thus higher usage intensity gender ratio. As indicated in Fig. 6, age groups 13–16 and 14–17 years have higher DAUs to MAUs ratios than the other age groups thus, girls who are 13–17 years old are more likely to spend more time on the platform. The more time that users spent on the platform, the more Instagram or Facebook assigns interest for users. For example, if we have two users (User A and User B) within the same age group, but User A spends more time on Instagram than User B. Then, Instagram is more likely to assign more interests to user A. These assigned interests are possibly false positive and do not accurately represent users’ actual interests. Facebook and Instagram use machine learning algorithms to infer interests about its users (https://www.facebook.com/business/news/good-questions-real-answers-how-does-facebook-use-machine-learning-to-deliver-ads). Users who spend more time on the platform are also more expected to engage more on the platforms (*i.e*., like and comment on posts). The more interactions on the platform, the more likely the algorithm is run again and again to assign interest to users. For instance, users who are 13 years old are more engaged on the platform, so Instagram picks more interest for them related to both STEM and Placebo. These teen users are expected to be targeted based on interests that do not reflect their actual interests. This is why we saw that for interests in *Science* and interests in placebo, the GGIs for age groups 13–16 and 14–17 years are higher than the GGIs for the other older age groups. In all likelihood, this stipulates what we are measuring on Instagram is time spent on the platform and usage intensity and, not actually interest related to STEM.

### Science hashtag on instagram

**Figure 7:**
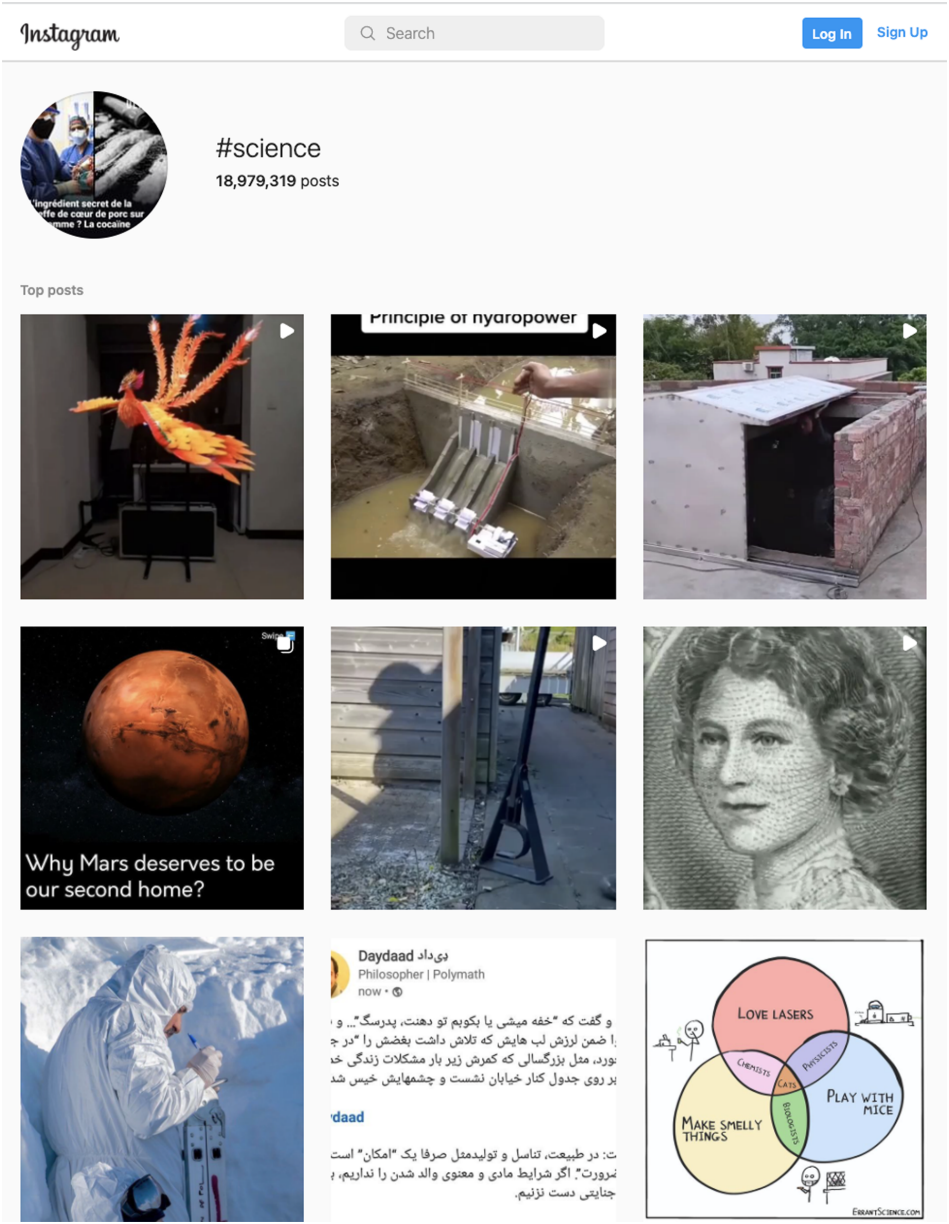
The hashtag #science on Instagram.

**Figure A5:**
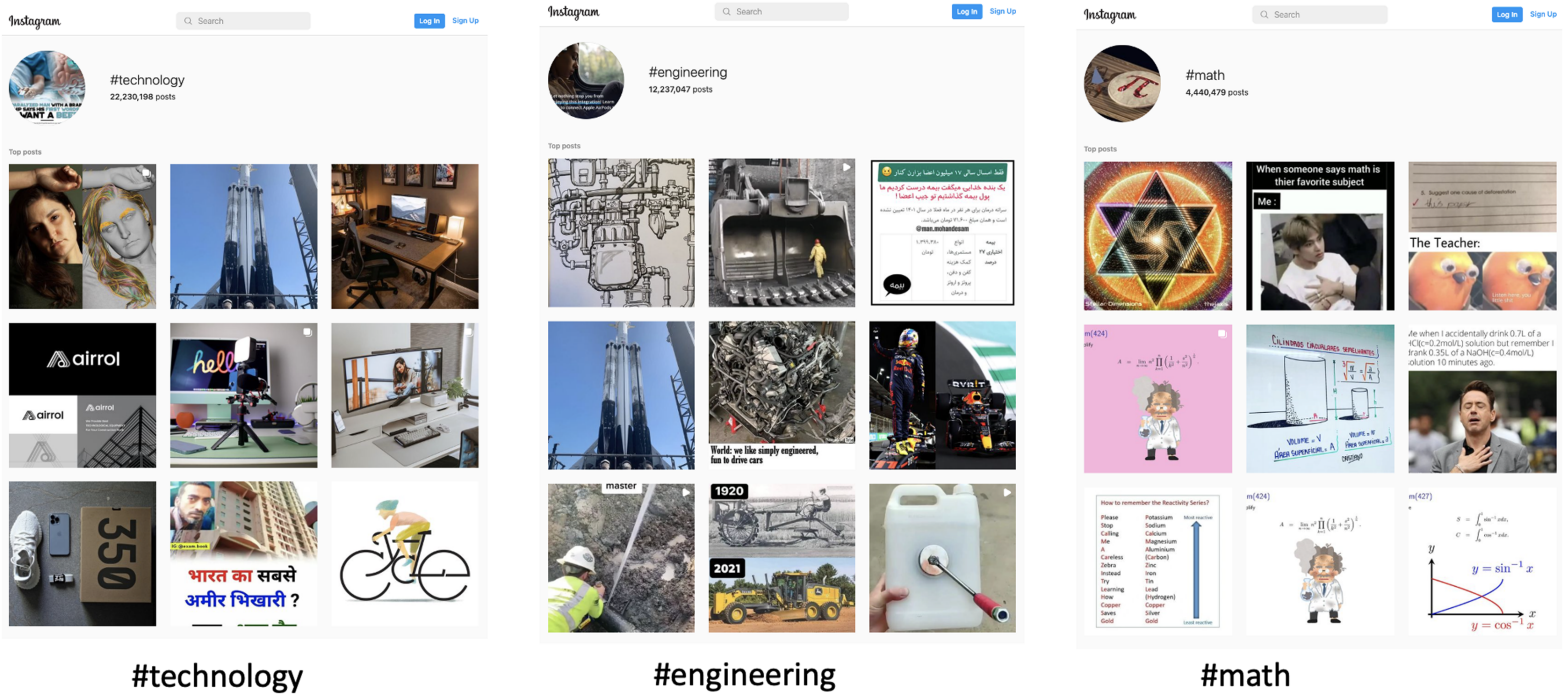
The hashtags #technology, #engineering, and #math on Instagram.

In order to explore what is classified as *Science* on Instagram, we also looked at pictures under the hashtag #science. Figure 7 shows that there is a variety of things covered under the *Science* hashtag on Instagram. These things could be directly related to science (*e.g*., pictures illustrating the principles of hydro-power) and unrelated to science (*e.g*., images of a door and roof). This raises many questions about how Instagram defines *Science* and how it assigns interests related to science to users. This also suggests that what we are measuring on Instagram is not necessarily related to STEM or the leaky pipeline. We also inspected pictures under the hashtags #technology, #engineering, and #math on Instagram (Fig. A5) to understand what might be classified as being related to an interest in STEM. As with #science, we found that pictures under those hashtags often do not appear to be related to STEM.

## Discussion

This article explores the use of Facebook and Instagram Ads audience estimates for modeling the gender-specific decline in interest in STEM across age, *i.e*., modeling one element of the leaky pipeline phenomenon. At the USA Metro level on Facebook, women, compared to men, are not losing interest in either *Science, Technology, Engineering*, and *Math* at older ages. On Instagram, however, the relative trend indicates that women, compared to men, are losing interest in *Science* at older ages. While this relative trend across the ages is in line with a leaky pipeline, the absolute levels observed are not: even at older ages, the data suggests higher levels of interest for women than for men. In order to understand this more, we assess the quality of Instagram data by introducing placebo baselines, and usage intensity gender ratio analysis. Among our findings, we show that interests in Placebo such as interest in *Music* display similar patterns as interest in *Science* across age groups on Instagram. Moreover, we show that teenagers (13–17) years old have higher usage intensity gender ratio than older users. As teens spend more time on the platform, this makes them vulnerable to being assigned more interests that are attached to their profiles. This could suggest that we are using the marketing APIs as a tool to measure activity level on the platform, and not interest in STEM. We also looked at pictures under the hashtag #science on Instagram. Despite that the hashtag covers a wide range of things that could be classified under *Science*, there are pictures that are not entirely related to Science or STEM in general. This indicates that it is hard to obtain a definition or classification of *Science* from Instagram. While it is known that, at a high level, Facebook and Instagram assign interests based on users’ publications and activities on the platform (https://www.linkedin.com/pulse/how-facebook-defines-interests-targeting-irina-yandulina), the details of the Instagram interest classification algorithm remain a black box. Facebook and Instagram do not only track users on their platform, but also track web behavior outside of their own platform. This includes tracking users across other websites and services, into the various apps they are using on their phone and to the places they physically visit in the real world—especially if they decide to check in on Facebook while they are there (https://www.wired.com/story/ways-facebook-tracks-you-limit-it/). For example, Aguiar et al. (2022) showed that Facebook can track 55% of the websites visited by Facebook users, and 44% of non-Facebook users, which amounts to 41% and 38% of browsing time, respectively. Therefore, profiling users is far beyond the science hashtag example given in the article. Recently, Apple released an update for iPhones with a new popup that asked users if they wanted to allow apps on their phones to target the user for ads (https://www.cnbc.com/2021/11/13/apples-privacy-changes-show-the-power-it-holds-over-other-industries.html). This change in Apple’s privacy could lead to the suffering of the Facebook Ads targeting. More important, similar patterns that are observed between interests in Science and interest in Placebo or usage intensity warrant extra caution in the use of Marketing APIs as a source of social interest.

Facebook advertisement data that are filtered by interests have shown promise in measuring schizophrenia awareness (Saha et al., 2017), investigating culture at the country, subnational, and local levels (Obradovich et al., 2020), assessing how the Brazilian culture is distributed around the world (Vieira et al., 2020), and exploring the cultural assimilation of Mexican Immigrants (Stewart et al., 2019). In spite of that, this study shows that there is no strong evidence to conclude that online advertisement data that are filtered by interests can be used to study the gender gap in STEM in the USA. These interests in STEM do not reflect users’ actual interests and are more prone to be false positives. This study contributes to previous work by not only combining Facebook and Instagram advertisement data to look at the gender gap in STEM in the USA, but also evaluating these novel online sources and comparing them to placebo and usage intensity gender ratios.

Still, it may be too early to give up on this potentially rich data source. Our effort is restricted to specific interests in STEM; we acknowledge that it is impossible to generalize the limitations we have found without future studies. Future work, for example, could look at partitioning the data based on the size of the US metro and comparing the gender gap index across age groups accordingly. Furthermore, to assess the accuracy of interest assignment, one could use the advertising platform to run a survey assessing the interests of the reached audience directly. The DAUs and MAUs change over time, with DAUs changing several times within 24 h and MAUs typically changing every 1–2 weeks. This is both a strength, in terms of recency, as well as a limitation, in terms of time-dependency of the analysis. Furthermore, the algorithms used to infer user attributes can occasionally change, which can make it hard to obtain consistent and comparable signals over time. For example, (Palotti et al., 2020) observed a sudden change in March 2019 in how Facebook classifies users by countries they have lived in. Unfortunately, Facebook does not provide access to historical data to show changes over time. Therefore, long-term effort is required to monitor the data with respect to seasonal variation across, say, 1 year. Finally, replicating our analysis using placebo interests in other countries could help with understanding whether interest-specific, rather than usage-related, conclusions can be drawn in other contexts.

We hope that this work will encourage future efforts to use our methodology to gather user interest from Facebook and Instagram for other use cases and research problems. We also hope that the results presented here will encourage future researchers to test the face validity of these potentially rich data sources.

## Additional Information and Declarations

### Data Availability

The following information was supplied regarding data availability: The data and code are available at figshare: Al Tamime, Reham (2022): PeerJ Article Supplemental Files. figshare. Dataset. https://doi.org/10.6084/m9.figshare.19561288.v1.
